# Supplementary material for: Role of neutrophil extracellular traps in radiation resistance of invasive bladder cancer
Source: Nat Commun. 2021 May 13;12:2776. doi: 10.1038/s41467-021-23086-z (PMC8119713; doi:10.1038/s41467-021-23086-z)
Supplement: Supplementary file 1 — Supplementary Information [file 41467_2021_23086_MOESM1_ESM.pdf]

SUPPLEMENTARY INFORMATION

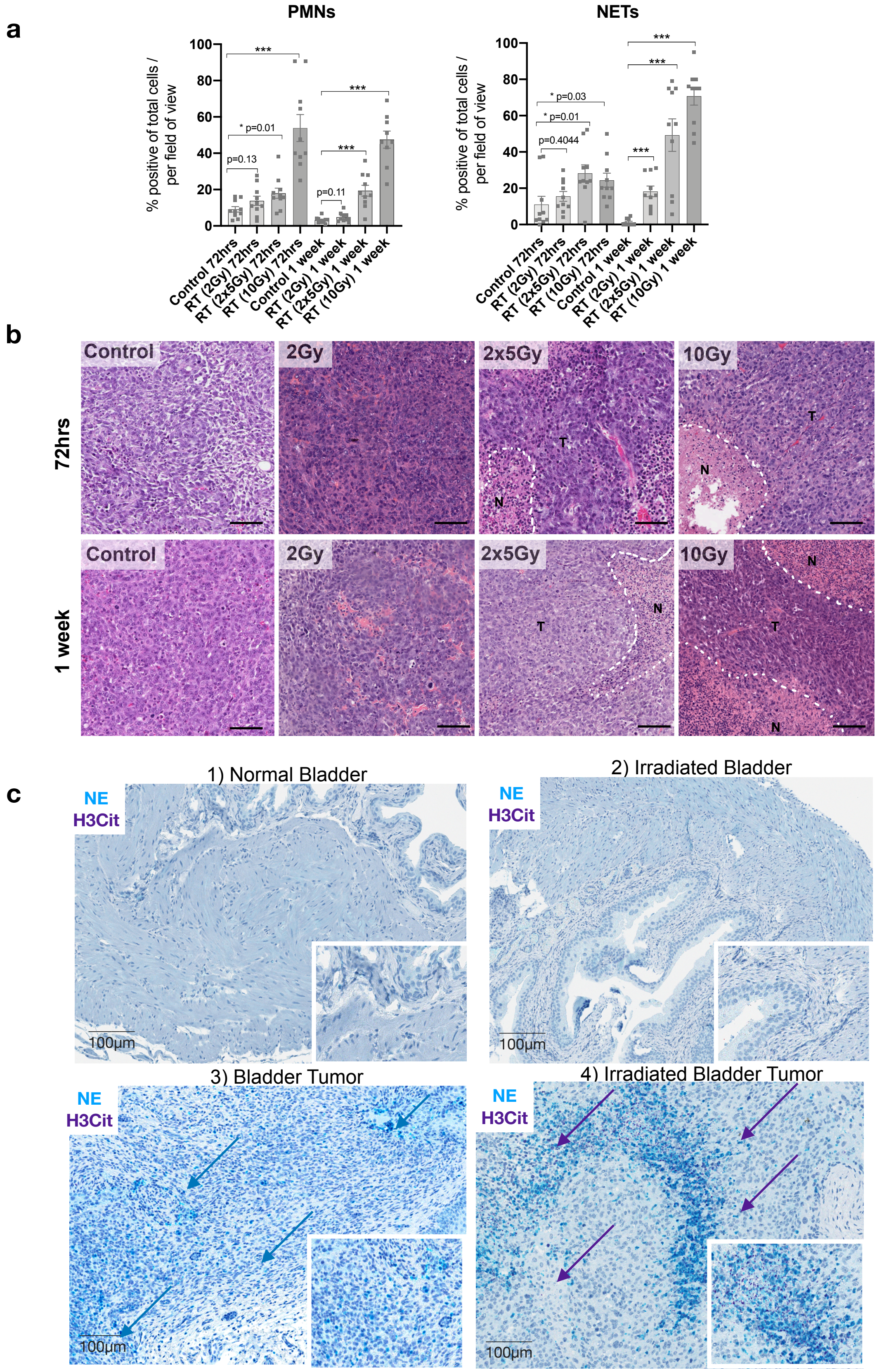

Supplemental Figure 1: (A) Quantification of PMNs and NETs in tumors of non-irradiated controls or tumors radiated with 2Gy, 2x5Gy or 10Gy 72 hrs or 1 week post-RT (n=10 mice per arm, N=3), data represented as mean± SEM, unpaired two tailed student t-test was used to assess statistical significance (*p<0.05, ***p<0.001) (B) Representative H&E of non-irradiated controls and irradiated tumors (2Gy, 2x5Gy, 10Gy) 72h or 1 week post-RT. Tumor region (T), Necrosis region (N), scale bar 72µm (C) Representative IHC images of normal murine bladder, irradiated bladder (10Gy), bladder with MB49 tumor, and irradiated bladder tumor (10Gy) stained with NE-blue and H3Cit (purple), scale bar 100µm. Images in (B) and (C) are representative images from 3 independent experiments.

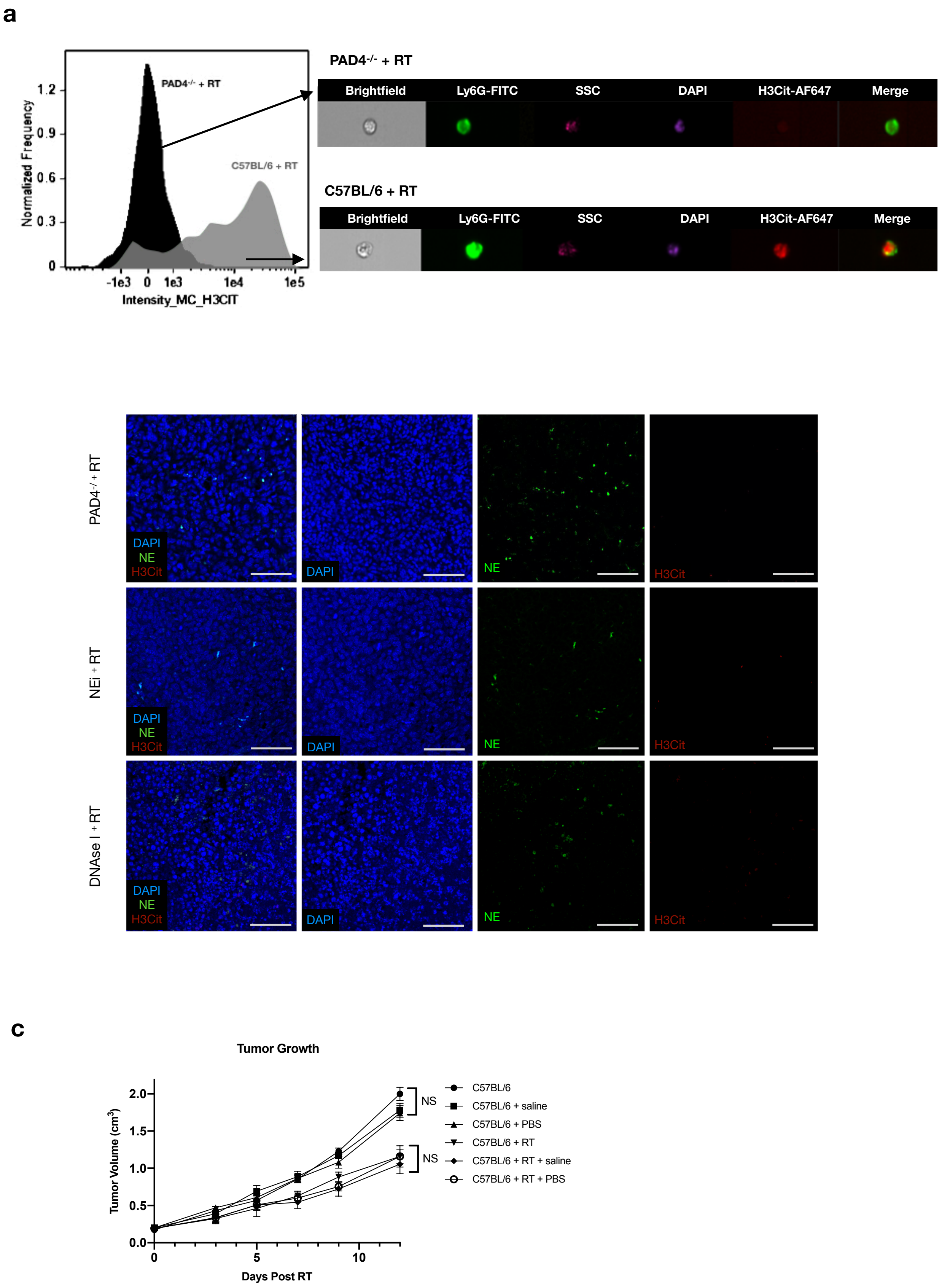

Supplementary Figure 2: (A) Image Stream Flow Analysis of PAD4$^{-/-}$ tumors confirming the absence of NETs in these tumors marked by negative H3Cit staining (n=7 mice per group) (B) Representative confocal images from 3 independent experiments of PAD4$^{-/-,}$ C57BL/6 + NEi and C57BL/6 + DNAse I tumors 11 days post-RT stained for PMNs and NETs (NE-green, H3Cit-red), DAPI (blue), 20x, scale bar 50μm (C) Tumor growth kinetics of C57BL/6 control mice treated with PBS or saline (n= 8 mice per group, N=3). Data represented as mean± SEM, two-way ANOVA with Bonferroni's multiple comparison's test was used to assess statistical significance, NS= non-significant (p>0.05)

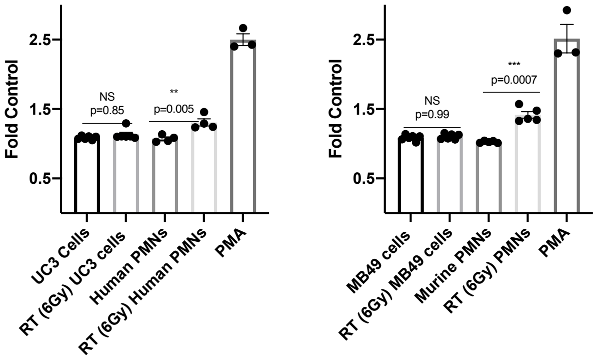

Supplementary Figure 3: *In vitro* Sytox fluorescence of irradiated UC3 cells, MB49 cells and PMNs. UC3 or MB49 cells were seeded in a 96-well plate and were irradiated 6Gy. 3hrs later the plate was read in a microplate reader to measure Sytox Fluorescence. Data represented as fold control from 3 independent experiments. Data represented as mean± SEM, one-way ANOVA with Tukey's multiple comparisons test was used to assess statistical significance, NS= not significant.

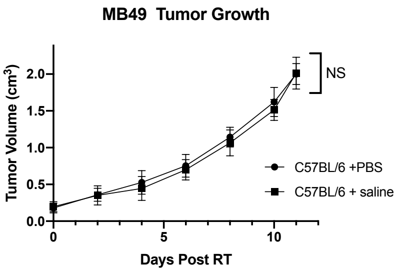

Supplementary Figure 4: MB49 tumor growth with vehicle treatment. C57BL/6 mice were injected s.c in the right flank with MB49 (500,000 cells). Mice were administered PBS (i.p) or saline (i.m) as controls (n=8 mice per group). Data represented as mean± SEM, two-way ANOVA was used to assess statistical significance, NS= not significant p>0.05

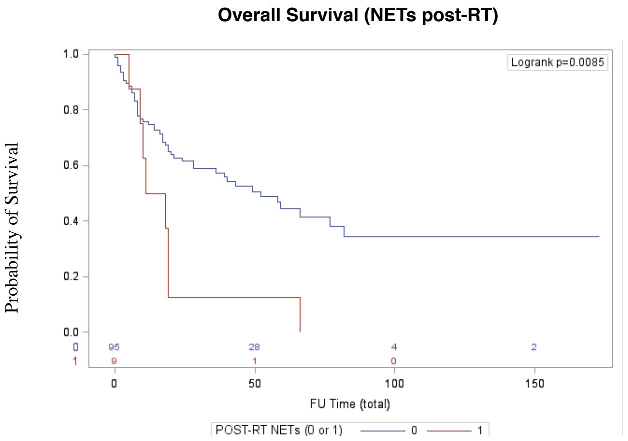

Supplementary Figure 5: Overall survival of patients with NETs in tumor post-RT (n=104 patients)**,** log rank (Mantel-cox) test was used to assess statistical significance **p<0.01

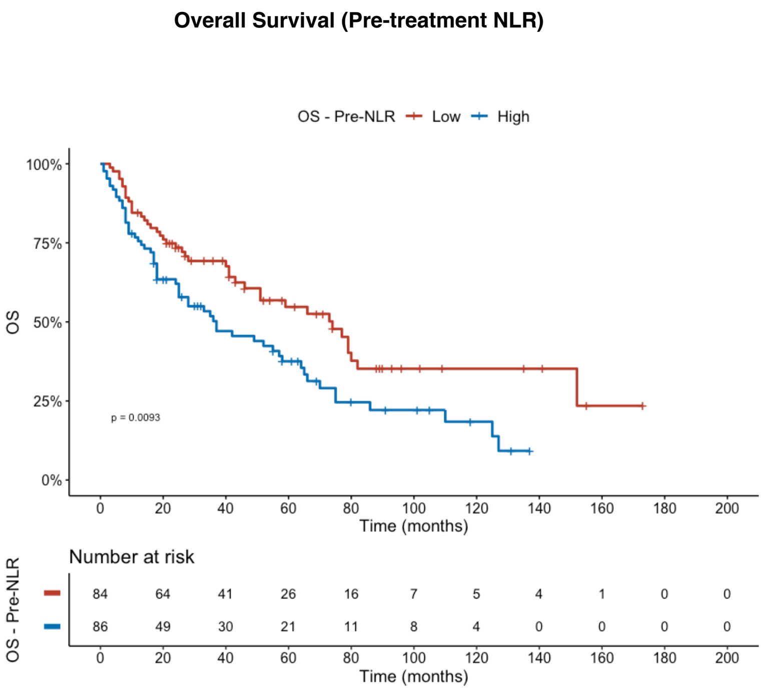

Supplementary Figure 6: Overall survival of MIBC RT-cohort (n=170 patients) with high pre-treatment NLR (≥2.8) or low pre-treatment NLR (<2.8) determined by the median, log rank (Mantel-cox) test was assess statistical significance. MVA analysis for cohort (HR 1.75, 95%CI 1.13-2.73, p=0.013)
