# Supplementary material for: Role of neutrophil extracellular traps in radiation resistance of invasive bladder cancer
Source: Nat Commun. 2021 May 13;12:2776. doi: 10.1038/s41467-021-23086-z (PMC8119713; doi:10.1038/s41467-021-23086-z)
Supplement: Supplementary file 3 — Reporting Summary [file 41467_2021_23086_MOESM3_ESM.pdf]

nature research

Corresponding author(s): Dr. Wassim Kassouf

Last updated by author(s): March 29 2021

Reporting Summary

Nature Research wishes to improve the reproducibility of the work that we publish. This form provides structure for consistency and transparency in reporting. For further information on Nature Research policies, see our Editorial Policies and the Editorial Policy Checklist.

Statistics

For all statistical analyses, confirm that the following items are present in the figure legend, table legend, main text, or Methods section.

n/a	Confirmed	
☐	☒	The exact sample size (*n*) for each experimental group/condition, given as a discrete number and unit of measurement
☐	☒	A statement on whether measurements were taken from distinct samples or whether the same sample was measured repeatedly
☐	☒	The statistical test(s) used AND whether they are one- or two-sided<br>*Only common tests should be described solely by name; describe more complex techniques in the Methods section.*
☐	☒	A description of all covariates tested
☐	☒	A description of any assumptions or corrections, such as tests of normality and adjustment for multiple comparisons
☐	☒	A full description of the statistical parameters including central tendency (e.g. means) or other basic estimates (e.g. regression coefficient) AND variation (e.g. standard deviation) or associated estimates of uncertainty (e.g. confidence intervals)
☐	☒	For null hypothesis testing, the test statistic (e.g. *F*, *t*, *r*) with confidence intervals, effect sizes, degrees of freedom and *P* value noted<br>*Give P values as exact values whenever suitable.*
☒	☐	For Bayesian analysis, information on the choice of priors and Markov chain Monte Carlo settings
☒	☐	For hierarchical and complex designs, identification of the appropriate level for tests and full reporting of outcomes
☒	☐	Estimates of effect sizes (e.g. Cohen's *d*, Pearson's *r*), indicating how they were calculated

Our web collection on statistics for biologists contains articles on many of the points above.

Software and code

Policy information about availability of computer code

Data collection	No software was used for data collection
Data analysis	GraphPad Prism software version 9 was used for statistical analyses. Multivariate analysis was performed using SAS software version 9.2. Immunofluorescence/immunohistochemistry analyses were quantified using the open-source software QuPath version 6 using the positive cell detection algorithm.

For manuscripts utilizing custom algorithms or software that are central to the research but not yet described in published literature, software must be made available to editors and reviewers. We strongly encourage code deposition in a community repository (e.g. GitHub). See the Nature Research guidelines for submitting code & software for further information.

Data

Policy information about availability of data

All manuscripts must include a data availability statement. This statement should provide the following information, where applicable:

- Accession codes, unique identifiers, or web links for publicly available datasets
- A list of figures that have associated raw data
- A description of any restrictions on data availability

The authors declare that the findings of this study are available within the paper and its supplementary files. Raw data are available from the corresponding author upon reasonable request.

Field-specific reporting

Please select the one below that is the best fit for your research. If you are not sure, read the appropriate sections before making your selection.

- [x] Life sciences
- [ ] Behavioural & social sciences
- [ ] Ecological, evolutionary & environmental sciences

For a reference copy of the document with all sections, see nature.com/documents/nr-reporting-summary-flat.pdf

Life sciences study design

All studies must disclose on these points even when the disclosure is negative.

Sample size	No statistical method was used to predetermine sample size. In vitro experiments were performed using biological and technical triplicates. In vivo experiments were performed using a minimum sample size of n=8 mice per arm as this is expected to have >80% power to detect a 25% reduction in tumor volume at a p-value of 0.05 as previously published (Srivastava et al., 2016, Rompre-Brodeur et al., 2019). For immunofluorescence analyses, multiple fields of view per mice were quantified, and exact numbers are specified in the figure legends. For the clinical data, samples were included based on tissue availability.
Data exclusions	Relevant data were not excluded
Replication	All findings reported were reproducible as data related to in vitro and in vivo experiments resulted from observations performed in three or more independent experiments with comparable results.
Randomization	In vitro experiments were performed using biological and technical triplicates. For in vitro experiment involving PMNs, cells were evenly seeded in triplicates into each experimental group from the same pool. For in vivo experiments, post tumor injection all mice were randomized into treatment and controls groups in a unbiased fashion. For clinical data, a multivariable logistic regression was performed to assess the role of each covariates on a complete response post-RT. In order to estimate the impact of each variable on overall survival a Cox model was performed. Log linearity hypothesis were verified for quantitative variables and proportional hazard assumptions were verified for all variables. Final multivariate model was created using a stepwise selection method. Statistical significance was set at 5%. (Table 2)
Blinding	Blinding was not applicable for in vivo experiments as mice were receiving different treatments. For the retrospective patients IHC experiments, staining and expression of markers was blinded to clinical outcome. Additionally TMA cores were analyzed using a script to ensure unbiased and homogeneous quantification of data. Immunofluorescence images were analyzed in a blinded manner - as experimental groups were unknown while capturing microscope images and data was analyzed using an automated script and all detections were re-verified.

Reporting for specific materials, systems and methods

We require information from authors about some types of materials, experimental systems and methods used in many studies. Here, indicate whether each material, system or method listed is relevant to your study. If you are not sure if a list item applies to your research, read the appropriate section before selecting a response.

Materials & experimental systems

n/a	Involved in the study	
	x	Antibodies
	x	Eukaryotic cell lines
x		Palaeontology and archaeology
	x	Animals and other organisms
	x	Human research participants
x		Clinical data
x		Dual use research of concern

Methods

n/a	Involved in the study	
x		ChIP-seq
x		Flow cytometry
x		MRI-based neuroimaging

Antibodies

Antibodies used	Mouse antibodies (IF/IHC)<br>H3Cit (Cat#: ab5103, 1:100, Abcam), Ly6G (Cat#: 551459, 1:100, BD Biosciences), CD8 (Cat#: 98941, 1:100, Cell-Signaling Technologies), NE (Cat#: Bs-698-2R, 1:100, Bioss USA), NE (Cat#: Bs-698-2R-AF488, 1:100, Bioss USA), H3Cit-AF568 (ab5103, was conjugated to AF568 Cat#: A-20184, 1:100 Thermo Fisher Scientific), AF488 (Cat#: A-11006, 1:1000, Thermo Fisher Scientific)<br><br>Human antibodies (IF/IHC):<br>NE (Cat#: MAB9167SP, Clone: 950334, 1:100, Novus Biologicals), CD8 (Cat#: 790-4460, Clone: SP57, 1:100, Roche Diagnostics)<br><br>Multiplex-IHC :<br>Perkin Elmer (Cat#: NEL810001KT) using fluorophores Opal 520, Opal 570, Opal 620

	Imaging Flow Cytometry:<br>Ly6G-FITC (Cat#: 11-9668-82, Clone 1A8, 1:200, Thermo Fisher Scientific), H3Cit-AF647 (ab5103 conjugated to AF647, 1:100).
Validation	Antibody validation was provided by the company, all antibody dilutions were optimized prior use.<br>Mouse antibodies (IF/IHC)<br>H3Cit (Cat#: ab5103, 1:100, Abcam) was validated for NETs detection by IF in this study (Brinkmann et al, Front Immunol. 2016)<br>Ly6G (Cat#: 551459, 1:100, BD Biosciences) : https://www.bdbiosciences.com/ca/reagents/research/antibodies-buffers/immunology-reagents/anti-mouse-antibodies/cell-surface-antigens/purified-rat-anti-mouse-ly-6g-1a8/p/551459<br>CD8 (Cat#: 98941, 1:100, Cell-Signaling Technologies): https://www.cellsignal.com/products/primary-antibodies/cd8a-d4w2z-xp-rabbit-mab-mouse-specific/98941<br>NE (Cat#: Bs-698-2R, 1:100, Bioss USA) and NE (Cat#: Bs-698-2R-AF488, 1:100, Bioss USA) https://www.fishersci.com/shop/products/neutrophil-elastase-ela2-mouse-anti-human-clone-950334-novus-biologicals-2/p-7105341<br>AF488 (Cat#: A-11006, 1:1000, Thermo Fisher Scientific) : https://www.thermofisher.com/antibody/product/Goat-anti-Rat-IgG-H-L-Cross-Adsorbed-Secondary-Antibody-Polyclonal/A-11006<br><br>Humans antibodies (IHC):<br>NE (Cat#: MAB9167SP, Clone: 950334, 1:100, Novus Biologicals): https://www.fishersci.com/shop/products/neutrophil-elastase-ela2-mouse-anti-human-clone-950334-novus-biologicals-2/p-7105341<br>CD8 (Cat#: 790-4460, Clone: SP57, 1:100, Roche Diagnostics) : http://ww.ventanadiscovery.com/product/33?type=28<br><br>Multiplex-IHC<br>Perkin Elmer (Cat#: NEL810001KT) using fluorophores Opal 520, Opal 570, Opal620: https://www.perkinelmer.com/lab-solutions/resources/docs/DTS_1-05-40-NR-OPALGUIDELINES_Opal4-7-color_Manual_Kit_Insert.pdf<br><br>Imaging Flow Cytometry:<br>Ly6G-FITC (Cat#: 11-9668-82, clone 1A8, 1:200, Thermo Fisher Scientific): https://www.thermofisher.com/antibody/product/Ly-6G-Antibody-clone-1A8-Ly6g-Monoclonal/11-9668-82<br>H3Cit-AF647 (ab5103 conjugated to AF647) was validated for imaging flow cytometry in the study by (Croker et al, Science Signalling. 2018)

Eukaryotic cell lines

Policy information about cell lines

Cell line source(s)	UM-UC3 cell line was obtained from Sigma Aldrich. MB49 cell line were a gift from Dr. Peter Black (University of British Columbia - https://www.emdmillipore.com/CA/en/product/MB49-Mouse-Bladder-Carcinoma-Cell-Line,MM_NF-SCC148).
Authentication	Cell line authentication was performed for the UM-UC3 cell line by Sigma Aldrich. Murine cell line MB49 was maintained as a frozen stock. Cell lines were not cultured more than 4 weeks prior experiments to ensure cell line authenticity and phenotype stability.
Mycoplasma contamination	All cell lines tested negative for mycoplasma contamination as required by the Research Institute McGill University Health Center (RI-MUHC)
Commonly misidentified lines<br>(See ICLAC register)	No commonly misidentified cell lines were used in the study.

Animals and other organisms

Policy information about studies involving animals; ARRIVE guidelines recommended for reporting animal research

Laboratory animals	Seven to ten week old male C57BL/6 mice and athymic C57BL/6 mice were obtained from Charles River Laboratories. Peptidyl arginine deiminase type IV knockout (PAD4-/-) mice were a gift from Dr. Allan Tsung (The Ohio State University, USA). Tlr4tm1.2Karp (TLR4-/-) mice were obtained from the Jackson Laboratory (Stock#029015).
Wild animals	N/A. The study did not involve wild animals
Field-collected samples	N/A. The study did not involve field-collected samples
Ethics oversight	All animal experiments were performed according to the Canadian Council on Animal Care. All procedures were approved by the McGill University Animal Care Committee (Protocol #7585) at our facility.

Note that full information on the approval of the study protocol must also be provided in the manuscript.

Human research participants

Policy information about studies involving human research participants

Population characteristics	Clinico-pathological characteristics of the patient population are described in Table 1 of the manuscript.
Recruitment	Our retrospective analyses were conducted on a cohort of muscle-invasive bladder cancer patients that were treated with radiation based therapy at our institution. Pre-treatment tumor biopsies and routine bladder biopsy samples 1-month post

	radiation were collected stored and used under IRB approval. Covariates were controlled for by performing a multivariable analysis (Table 2 of manuscript).
Ethics oversight	All patient samples were collected with informed consent and ethics approval was obtained (REB- RI-MUHC #2017-2612)

Note that full information on the approval of the study protocol must also be provided in the manuscript.
